# PREPARING THE NURSING WORKFORCE TO CARE FOR OLDER ADULTS IN THE 21ST CENTURY

**DOI:** 10.1093/geroni/igac059.209

**Published:** 2022-12-20

**Authors:** Deborah Brabham

**Affiliations:** Florida State College at Jacksonville, Jacksonville, Florida, United States

## Abstract

Nurses entering the workforce may have limited education in gerontological nursing. Therefore, many nurses are unprepared to provide quality care to older adults. An unprepared nursing workforce could negatively influence older adults’ health outcomes. This study aimed to determine differences in senior nursing students’ knowledge, attitudes, and perceived competency about older adults based on enrollment in a bachelor of science in nursing degree (BSN), associate’s degree nursing (ADN), and practical nursing (PN) program that offers geriatric content in curricula. Albert Bandura’s social cognitive, triadic reciprocal determinism model provided the theoretical framework to underpin this research study. A non-experimental, descriptive survey design included a convenience sample of students enrolled in a BSN, ASN, and PN program. A total of 178 students participated in the study. Palmore Facts on Aging Quiz 2, Kogan’s Attitudes Toward Old People Scale, and the Hartford Geriatric Nurse Competency tool was used to collect data. From the results, it is clear that students enrolled in BSN, ADN, and PN programs demonstrated limited knowledge about facts on aging. Students’ attitudes toward older adults were positive, and a correlation was found between knowledge and attitudes. Students perceived competency about older adults was high but purely subjective. Students’ preference to work with older adults post-graduation in the PN group was higher than students in the BSN and ADN groups. These findings underscore the need to systematically design an evidence-based curriculum inclusive of geriatric content across (BSN, ADN, and PN) programs to prepare the nursing workforce to care for older adults.

